# Targeting tumor-infiltrating tregs for improved antitumor responses

**DOI:** 10.3389/fimmu.2024.1325946

**Published:** 2024-03-04

**Authors:** Diyuan Qin, Yugu Zhang, Pei Shu, Yanna Lei, Xiaoyu Li, Yongsheng Wang

**Affiliations:** ^1^ Cancer Center, Clinical Trial Center, West China Hospital, Sichuan University, Chengdu, Sichuan, China; ^2^ Cancer Center, National Medical Products Administration Key Laboratory for Clinical Research and Evaluation of Innovative Drugs, West China Hospital, Sichuan University, Chengdu, Sichuan, China; ^3^ Division of Thoracic Tumor Multimodality Treatment, Cancer Center, West China Hospital, Sichuan University, Chengdu, Sichuan, China

**Keywords:** regulatory T cells, immunotherapy, cancer, microenvironment, metabolism, checkpoint inhibitor, cytokine, chemokine

## Abstract

Immunotherapies have revolutionized the landscape of cancer treatment. Regulatory T cells (Tregs), as crucial components of the tumor immune environment, has great therapeutic potential. However, nonspecific inhibition of Tregs in therapies may not lead to enhanced antitumor responses, but could also trigger autoimmune reactions in patients, resulting in intolerable treatment side effects. Hence, the precision targeting and inhibition of tumor-infiltrating Tregs is of paramount importance. In this overview, we summarize the characteristics and subpopulations of Tregs within tumor microenvironment and their inhibitory mechanisms in antitumor responses. Furthermore, we discuss the current major strategies targeting regulatory T cells, weighing their advantages and limitations, and summarize representative clinical trials targeting Tregs in cancer treatment. We believe that developing therapies that specifically target and suppress tumor-infiltrating Tregs holds great promise for advancing immune-based therapies.

## 1 Introduction

In recent years, novel therapies such as immunological checkpoint inhibitors (PD-1, CTLA-4), tumor dendritic cell (DC) vaccines, chimeric antigen receptor T (CAR-T) cells, TCR-T cells, and tumor-infiltrating lymphocytes (TILs) have achieved notable therapeutic success by amplifying the antitumor activities of effector cells (1–6). The tumor microenvironment (TME) is multifaceted, containing both antitumor immune cells and immune-inhibitory components like regulatory T cells (Tregs), myeloid-derived suppressor cells (MDSCs), tumor-associated macrophages, and tumor-associated fibroblasts (7–9). Therapies targeting immune-suppressive cells also hold significant potential in cancer treatment (10–13). This review will primarily delve into immunotherapies targeting intratumoral Tregs (Ti-Tregs) and their future prospects.

Treg cells are widely distributed throughout the body, not just in lymphoid organs, but also in the lungs, intestines, mucosal skin, and notably, within the tumor microenvironment (14). Tregs possess a bifunctional role in immunity. On one hand, they can maintain immunological homeostasis inside the body and avoid excessive immune activation. Deficiency or malfunction of Tregs may lead to autoimmune diseases (15). Treg cells, on the other hand, have immunosuppressive functions in cancer. Multiple evidence demonstrate that Tregs not only reduce the antitumor activity of effector T cells (Teff) in the TME, but also suppress DCs and macrophages (16–18). As a result, eliminating or inhibiting Ti-Treg cells in cancer patients may improve the effectiveness of immunotherapy against malignancies (10). Determining how to efficiently and selectively target or suppress tumor-infiltrating Tregs is an important research topic. This review focuses on the phenotypic and functional distinctions among Treg subgroups, as well as the mechanism by which Tregs suppress immunological responses. We also review and analysis the features of various Tregs- targeting immunotherapy strategies, as well as the possibilities for future treatments.

## 2 Characteristics and subpopulations of Tregs

Gershon and Kondo revealed half a century ago that T cells in mice not only operate as effectors, but also dampen the immune response (19). Mice approximately three days old undergoing thymectomy, leading to partial dysfunction of T cells, suffer a variety of autoimmune disorders (20). Building upon this, Sakaguchi and colleagues identified the CD25 molecule (IL2 receptor alpha chain) within this suppressive T cell population, thereby better characterizing such unique subset. Notably, the autoimmune disorders in thymectomized mice were reduced when they were reinfused with CD25-expressing CD4 T cells (21).

Following CD25 discovery, the identification and research of the transcription factor Foxp3, a crucial regulator in the formation and function of Tregs, marked another significant milestone in Treg research (22–25). Mutations in the Foxp3 gene, even just a two-base insertion, result in Scurfy mice. These mice, with impaired Treg production and function, display severe autoimmune reactions, including significant skin inflammation, enhanced T cell activity, and a marked expansion of T cells in both spleen and lymph nodes (22, 26). Similarly, Foxp3 gene mutation in humans could also lead to Tregs impairment and thus severe systemic autoimmune diseases such as IPEX syndrome (Immune Dysregulation, Polyendocrinopathy, Enteropathy, X-linked syndrome), whose clinical manifestations were first reported in 1982 (27).

Hence, CD25 and Foxp3 double positivity served as identification markers for the Treg population. However, the functionality and surface markers among CD4+ regulatory T cells, identified by CD25+Foxp3+, exhibit highly heterogeneous. For instance, some conventional effector T cells also transiently express CD25 and Foxp3 after activation by TCR signaling, which can still secrete IL-2 and IFN-γ without immunosuppressive effects (28, 29).

The expression of CD127 is negatively correlated with the suppressive function of Treg and the expression of FOXP3, some research teams suggested using CD127 (the alpha subunit of the IL-7 receptor) in combination with CD25 and CD4 as markers for human Tregs (30, 31). However, naïve T cells can also transiently express low levels of foxp3 and CD127 after activation by TCR signals (32), Consequently, this approach might face challenges in effectively distinguishing Tregs from certain activated conventional T cells (Tconvs).

To further distinguish Tregs subpopulations, Sakaguchi et al. subdivided Treg cells into natural Treg cells (nTreg) and peripheral Treg cells (pTreg) based on their origin and suppressive activity (16). Among them, nTregs, which are also known as thymic Tregs (tTregs), primarily derive from the thymus, and the main function of nTreg is to maintain immunological homeostasis and autoimmune tolerance. As previously indicated, after thymectomy in mice, nTregs are reduced, and autoimmune disorders occur spontaneously. pTregs evolve from peripheral Tconvs under the influence of TCR signaling and TGF-β stimulation (33, 34). Due to their distinct origins, nTregs and pTregs exhibit significant differences in their TCR repertoires. nTregs, developing in the thymus under the influence of self-antigens, have a TCR repertoire skewed towards self-antigens (35, 36). In contrast, pTregs, derived from peripheral Tconv cells, exhibit a TCR repertoire more similar to Tconv cells, offering greater diversity. TCR repertoire analysis shows that both nTreg and pTreg contribute to the TCR repertoire of intratumoural Treg (37).

Furthermore, researchers have cocultured mouse CD4+ T cells together with retinoic acid or TGF-β *in vitro* and obtained Foxp3-positive Treg cells, namely, induced Tregs (iTregs), which is also called pTreg in some other studies (34, 38, 39).

To more accurately characterize the immunosuppressive status of regulatory T cells, researchers thus utilized CD45RA, CD25, and Foxp3 with different expression intensities to characterize the functional subpopulations of Tregs, namely, naïve or resting Tregs, effector Tregs (eTregs), and non-Tregs (10, 40). Naïve Tregs originate from the thymus, have not been activated by TCR, have a phenotype of CD45RA+CD25^LOW^FOXP3^LOW^, and exhibit minimal immunosuppressive ability. However, once these naïve Tregs migrate to the peripheral tissues and contact antigens in draining lymph nodes, they proliferate and differentiate into effector Tregs (eTregs). These eTregs are prevalent in most solid tumors (41), exerting a powerful immunosuppressive effect and characterized by a CD45RA-CD25^high^FOXP3^high^. It is worth noting that for most solid tumor types, higher intratumoral FOXP3 expression correlates negatively with patient prognosis (42–44). However, in certain unique tumor types, such as colorectal cancer as well as few head and neck tumors (45–47), heightened intratumoral FOXP3 expression indicates a more favorable prognosis. Subsequent studies into Tregs in these unique cases indicated that the predominant phenotype of FOXP3+ CD4+ T cells among such colorectal cancers was CD45RA-CD25^LOW^FOXP3^LOW^, labeled as non-Tregs. These Tregs lack immunosuppressive effects but are capable of secreting inflammatory cytokines such as IFN-γ and IL-17 (46, 48), whose presence is associated with better outcomes for tumor patients.

It is worth noting that the relationship between Tregs and the prognosis of hematological malignancies is intricate as well. Researchers have extensively investigated Treg cells across various hematological malignancies. Due to the Treg heterogeneity, limitations in Treg detection methods and variations in patient tissue specimens, the impact of Tregs on disease prognosis varies (49). While in classical Hodgkin lymphoma tissues, the expression of FOXP3 alone may not serve as an independent prognostic factor, a multivariate analysis model including FOXP3, PD-1, and granzyme B demonstrates certain predictive value for the prognosis of classical Hodgkin lymphoma patients, with higher levels of FOXP3 expression often correlating with longer overall survival (50). Additionally, in follicular lymphoma (FL) and germinal center (GC) diffuse large B-cell lymphomas (DLBCL), a higher Treg cell count is linked to a favorable prognosis. In pediatric acute lymphoblastic leukemia patients, there is a weak negative correlation between Treg cells in the bone marrow and the percentage of primitive cells in the peripheral blood, but in chronic lymphocytic leukemia, an increased number of Tregs is associated with poorer outcomes (49, 51).

## 3 Immunosuppression mechanism of Tregs

### 3.1 Molecules associated with Treg immunosuppression

Tumor-infiltrating eTregs are activated and possess robust immunosuppressive properties (52), with high expression of molecules like CD25, CTLA4, PD-1, ICOS, LAG-3, TIGIT, and members of the TNF receptor superfamily, like GITR, 4-1BB, and OX-40 (53). Moreover, they express chemokine receptors, such as CCR4, CCR5 and CCR10, which facilitate chemotactic migration of Tregs into the tumor microenvironment (54). CTLA-4 is an important inhibitory function-related molecule, which is constitutively and highly expressed in Tregs and belongs to the same family as CD28. Ti-Tregs utilize CTLA-4 molecules with a higher affinity than CD28 to engage with the B7-1/B7-2 (CD80/CD86) ligand molecules on antigen-presenting cells (APCs), thereby competitively reducing the CD28 stimulation signal on Tconvs (55). Moreover, by virtue of CTLA4 molecules, Tregs hinder the maturation process of APCs, reducing CD80/86 expression and elevating inhibitory signaling on APCs, thereby dampening their ability to activate conventional T cells (56, 57), leading them to an anergic state with hyporesponsive to antigen stimulation.

In tumor, PD-1 molecules inhibit TCR and CD28 signaling, which causes Tconv dysfunction or exhaustion (58). PD-1 is also highly expressed in Tregs and exhibit inhibit ability in Treg activity. Antagonistic therapy against PD-1 promotes the activation and expansion of Treg cells (59, 60). For instance, mice autoimmune pancreatitis was alleviated by specifically knocking out PD-1 in Treg cells, which increased Treg suppression capacity (61). It is reported that in patients receiving targeted immune checkpoint therapies, particularly anti-PD-1 treatments, a subset of patients’ tumors exhibit unexpected rapid progression, known as hyperprogressive disease (HPD) (62), whose mechanism has not been fully elucidated. Several studies have found that HPD during PD-1 monoclonal antibody treatment is correlated with marked activation and proliferation of Tregs within tumor, whereas patients without HPD exhibit diminished eTreg proliferation (60, 63, 64). The precise role of PD-1 in Tregs requires further investigation to be fully utilized in Treg-targeting cancer treatment.

CD25, also known as the alpha subunit of the IL-2 receptor, is highly expressed on Tregs. This subunit, in conjunction with the beta (CD122) and gamma (CD132) subunits, forms the high-affinity IL-2 receptor predominantly found on Tregs. The IL-2 receptor on Tconvs, on the other hand, lacks the alpha subunit and is made up of solely the beta and gamma subunits. The trimeric configuration of IL-2 receptor allows Tregs to bind the IL-2 cytokine with significantly higher affinity, particularly in the TME, compared to that on Tconvs. The distinction in IL-2 receptor composition and affinity plays a crucial role in the functional differentiation and immune response modulation between Tregs and Tconvs. Tregs compete with conventional T cells and result in a relative shortage of IL-2 for Tconvs, which is crucial for their activation and proliferation processes (65, 66).

Other molecules such as lymphocyte-activation gene 3 (LAG-3), T cell immunoglobulin and mucin domain-containing 3 (TIM-3), T cell immunoreceptor with Ig and ITIM domains (TIGIT), inducible costimulator (ICOS), glucocorticoid-induced tumor necrosis factor-related receptor (GITR) and CD27 are unregulated on Ti-Tregs and contributed to their immunosuppressive activity in tumor treatment (67). By binding to MHC-II molecules with high affinity, LAG-3 plays a role in regulating the activation and proliferation of T cells (68). High expression of TIM-3 on Treg cells biases them towards an effector Treg (eTreg) phenotype, endowing them with more potent immunosuppressive capabilities to inhibit the proliferation and survival of CD8 T cells (69). Elevated TIGIT expression in Treg results in the transcription factor CEBPα overexpression, which in turn boosts the secretion of the soluble effector molecule fibrinogen-like protein 2 (Fgl2), facilitating the Treg-mediated selective suppression of pro-inflammatory Th1 and Th17 cells (70). Tregs expressing high levels of ICOS demonstrate significantly increased proliferative activity (71). In ICOS knock-out mice, the proportion of Tregs is substantially reduced compared to their wild-type counterparts. Furthermore, ICOS signaling plays a crucial role in enhancing Foxp3 transcription, thereby supporting the survival and augmenting the suppressive functionality of Tregs (72). GITR also promotes the survival and proliferation of Treg cells (73). The presence of CD27 on Treg cells plays a vital role in preserving the immune balance of peripheral CD8 T cells. Blocking CD27 signaling in combination with anti-PD-1 therapy has been shown to increase the number of CD8+ T cells within tumors and improve the CD8+ T cell/Treg ratio, thereby enhancing the anti-tumor efficacy (74).

V-domain immunoglobulin suppressor of T cell activation (VISTA) is also an immune checkpoint expressed on multiple cells such as T cells, myeloid cells and tumor cells. Specifically, VISTA is highly expressed in naïve CD4+ T cells and Treg cells, and it can increase FOXP3 expression. Treg proliferation can be inhibited by VISTA blockage (75).

All of the above-mentioned markers can serve as potential targets in strategies aimed at harnessing Tregs for anti-tumor therapy.

### 3.2 Immunosuppressive cytokines

Treg cells can also suppress immune reactions in TME by releasing anti-inflammatory cytokines such as IL-10, TGF-β, and IL-35. These cytokines suppress the activity of Teffs and APCs (76, 77).

TGF-β not only diminishes the cytotoxicity of Teffs and NK cells but also promotes the differentiation of peripheral naïve T cells into induced Treg cells (iTregs) (78–80). Furthermore, TGF-β augments the immunosuppressive function of Treg cells by activating Foxp3 (81). Consequently, it is feasible to directly target the TGF-β cytokine using blocking antibodies, or to inhibit the TGF-β receptor through antibodies or small molecule drugs, thereby disrupting the TGF-β signaling cascade and its facilitative function in Treg-mediated immunosuppression (82, 83).

In the tumor microenvironment, IL-10 and IL-35, primarily secreted by different Tregs subsets, are instrumental in modulating immune responses. IL-10, known for its ability to suppress the activity of effector T cells and antigen-presenting cells, plays a significant role in diminishing anti-tumor immunity. IL-35, another key cytokine, works in conjunction with IL-10 to induce an exhausted state in tumor-infiltrating lymphocytes and impairs the formation of T cell memory. However, IL-35-producing Tregs are vital for maintaining tolerance in auto-reactive T cells. Therefore, targeting IL-35 to inhibit Treg activity in cancer therapy necessitates careful consideration of potential autoimmune reactions (84).

Beyond cytokine secretions, Tregs can directly eliminate other effector immune cells by releasing cytotoxic components such as perforin and granzyme B (85).

### 3.3 Chemokines and chemokine receptors

Chemokines are also pivotal in the immunosuppressive process of Tregs. Treg cells exhibit high levels of chemokine receptors. Through chemotaxis like CCL17/CCL22-CCR4 (86, 87), CCL28-CCR10, CCL5-CCR5 et al. (88, 89), Tregs are recruited to the tumor microenvironment. For instance, CCR4 binds with ligands like CCL17 and CCL22 secreted by tumor cells, facilitating Treg infiltration (90). In a Pan02 pancreatic cancer mouse model, characterized by the secretion of the chemokines CCL17 and CCL22, the use of a CCR4 antagonist (CCR4-351) has shown significant effects in disrupting the CCR4-CCL17/CCL22 chemotactic axis, which effectively blocks the recruitment of Tregs to the TME (91).

In addition to chemotactic function, the CCR8-CCL1/CCL18 signaling axis, although not directly involved in the chemotactic aggregation of Tregs (which can chemotactically attract Th2 cells) (92, 93), is crucial for sustaining Treg homeostasis and immunosuppressive functions. Research suggests that the interaction between CCR8 and CCL1 enable the upregulation of FOXP3, IL-10, CD39 and granzyme B, resulting in proliferation and activation of Treg cells (94). Notably, CCR8 is highly expressed in Ti-Tregs but is rarely expressed by Teffs or naïve Tregs. Around 30-80% of Ti-Tregs possess CCR8 (95). Targeting intratumoral CCR8-positive Treg cells with an anti-CCR8 mAb could potentially induce tumor regression and promote lasting antitumor memory (95, 96).

### 3.4 Angiogenesis

Angiogenesis also influences the immunosuppressive activity of Ti-Tregs. Hypoxia can amplify the expression of CCL28, attracting CCR10-positive Treg cells into the tumor (88), suppressing the Teffs function, and also elevating the levels of VEGFA in the TME (97). When binding to its receptor VEGFR2, VEGFA suppresses the functions of APCs and Teffs (98–100), while also promoting the expansion and infiltration of Tregs within the TME (101, 102). VEGFA can further induce the expression of neuropilin 1 (NRP1) in Tregs, which promotes the aggregation of Tregs around tumor blood vessels, thereby promoting Treg aggregation around tumor blood vessels (103).

### 3.5 Metabolism adaptation

Tregs, utilizing their elevated expression of CD39 and CD73, which are also named ectonucleoside triphosphate diphosphohydrolase 1 (NTPDase1) and 5’-nucleotidase separately, to metabolize ATP and ADP within the tumor microenvironment into AMP. Subsequently, the CD73 molecule further converts AMP to adenosine (104, 105). Upon binding to the corresponding A2A receptors on Teffs, DC cells or NK cells, adenosine inhibits their activity and proliferation (106, 107). Furthermore, adenosine facilitates the proliferation of MDSCs and promotes the formation of M2 macrophages (108, 109), establishing an immunosuppressive microenvironment that attenuates the antitumor response (110).

Tregs also regulate metabolism within the tumor microenvironment through upregulating indoleamine 2,3-dioxygenase (IDO). IDO plays a crucial role in the metabolism of intermediate product tryptophan among the kynurenine pathway (111). IDO degrades tryptophan in the TME, resulting in Tonvs dysfunction (112). The engagement of CTLA-4 with CD80/CD86 lead to an further elevated expression of IDO in DCs (113). The consequential tryptophan reduction and kynurenine accumulation within the TME further induces exhaustion and compromised functionality of effector T cells.

It is worth mentioning that Tregs exhibit different metabolism preference and a superior metabolic adaptability compared to conventional T cells to sustain functional activity in tumor microenvironments characterized by hypoxia, glucose deficiency, lactic acid accumulation and the presence of various immunosuppressive metabolic products (114). Within the tumor microenvironment, tumor cells possess enhanced glycolytic capacity, leading to greater glucose consumption and consequent glucose restriction for TILs (115). Reduced glucose availability hampers mTOR activity, glycolytic function, and IFN-γ production in TILs, rendering TILs functionally ineffective (116). Through intrinsic regulation, Tregs demonstrate heightened Glut1 expression, glucose uptake, glycolysis rates, and fatty acid synthesis compared to Tconvs, preferentially utilizing glucose and fatty acids as substrates for energy production and biosynthesis (117, 118). Moreover, Foxp3 in Tregs was able to inhibit the expression of the Myc gene, enhancing oxidative phosphorylation and elevating the NAD : NADH ratio, leading to increased energy production (119).

Regarding lactate metabolism, Foxp3 was able to limit LDH’s reductive reaction from pyruvate to lactate, decreasing the production of NADH and promoting the oxidation reaction of L-lactate to pyruvate (119). Under high lactate conditions, Treg cells upregulate the expression of genes related to lactate metabolism, such as lactate dehydrogenase (LDH) and monocarboxylic acid transporter protein 1 (MCT1, lactate transporter protein) (120, 121), and are able to metabolize lactate as an alternative source of energy for the generation of intermediates needed for the tricarboxylic acid cycle (TCA) and phosphoenolpyruvate (PEP). Treg cells also highly express the CD36 molecule, a transporter protein for free fatty acids, which transports fatty acids and lipoprotein into the mitochondria. This protein shuttles fatty acids and lipoproteins to the mitochondria, facilitating mitochondrial energy metabolism and biological synthesis. This process alone with the peroxisome proliferator-activated receptor-β (PPAR-β) signaling pathway, support the survival and function of Ti-Treg cells (122). Such metabolic adaptations allow Tregs to better survive in environments with low glucose and high lactic acid or fatty acid levels, and they can resist the inhibitory effects of lactate on Treg function and proliferation.

However, it’s important to note that the immunosuppressive activity of Tregs also plays a critical role in maintaining immune balance and tolerance in the body. There is growing research interest in harnessing the suppressive functions of Tregs to reduce immune rejection in organ transplantation and to treat allergic or autoimmune diseases that are often caused by excessive immune activation (123–125). Treg can also be adopted to minimize immune-related adverse events (irAEs) in cancer immunotherapy. Scientists have developed pH-sensitive CTLA-4 antibodies, which bind to CTLA-4 on Treg cell surfaces and undergo internalization. In the acidic lysosomal environment, their affinity to CTLA-4 decreases, leading to dissociation. This process allows CTLA-4 to be recycled back to the Treg cell membrane, preserving the immunosuppressive role of Tregs in peripheral. Simultaneously, this antibody reactive exhausted Tconv cells, enabling their effector function. This approach, by preserving the peripheral immunosuppressive function of Tregs, mitigates irAEs while maintaining anti-tumor efficacy (126, 127).

**Figure 1:**
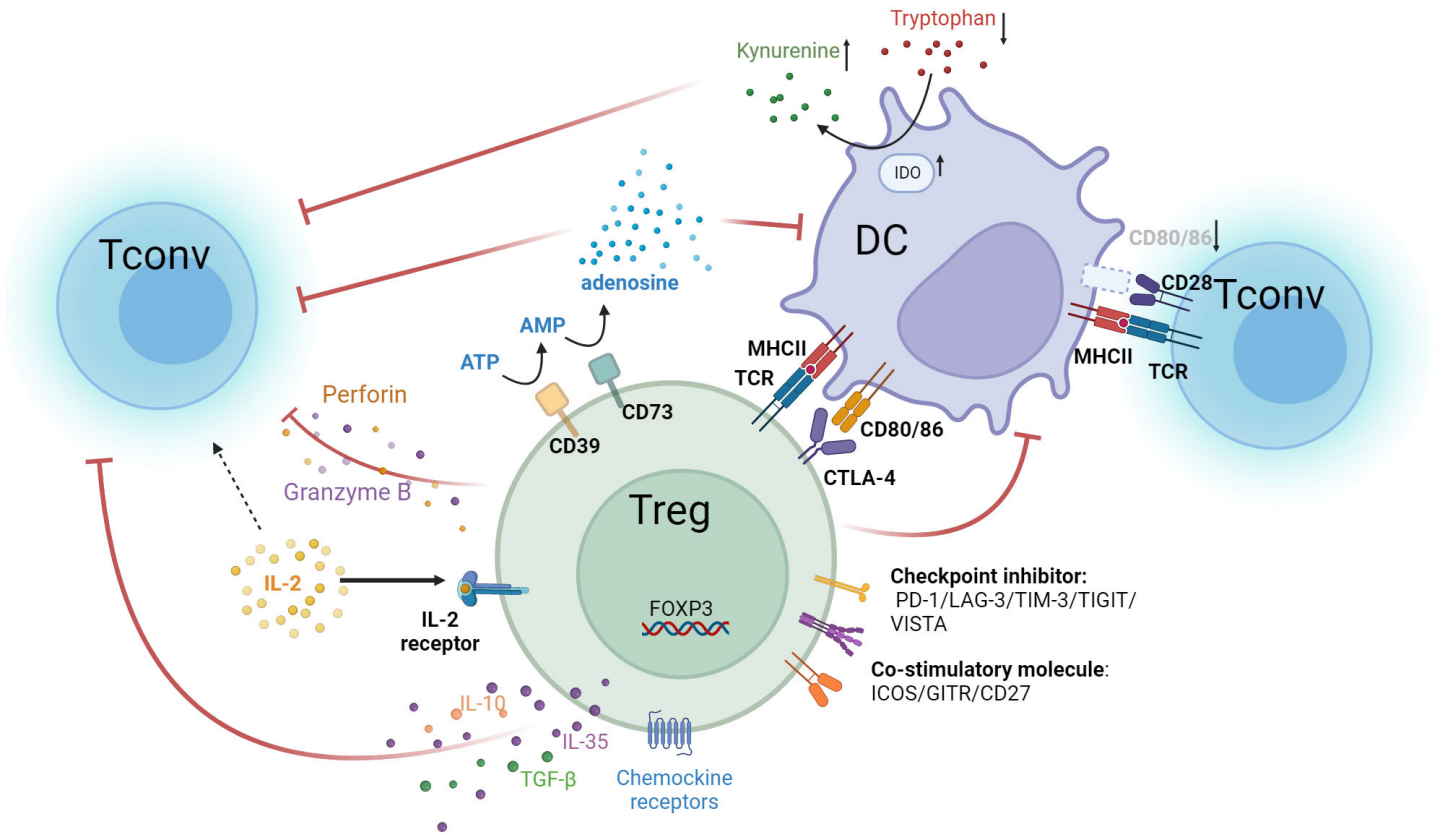
Multiple immunosuppressive mechanisms of Tregs. Treg cells express high-affinity IL-2 receptors, with CD25 as the α subunit of this receptor. In contrast, the IL-2 receptors on Tconv comprise only β and γ subunits and are of lower affinity. Tregs competitively bind IL-2 in the tumor microenvironment, causing a relative deficiency of IL-2 in Tconv and suppressing T cell effector functions. Tregs highly express CTLA-4 and bind to CD80/86 ligands on APCs. This not only inhibits APC function but also reduces the binding of CD80/86 expressed by APCs to CD28 molecules on T cells. As a result, the activation of Teff is attenuated. Additionally, suppression of DC cells by Tregs leads to overexpression of IDO, which decreases tryptophan and leads to kynurenine accumulation in the TME. This results in effector T cell exhaustion and dampens anti-tumor immunity. Tregs secrete immunosuppressive cytokines, such as IL-10, IL-35, and TGF-β, which inhibit the activity of T cells and APC cells. They also secrete perforin and granzyme B to directly lyse effector immune cells. Additionally, Tregs overexpress CD39 and CD73, converting ATP in the TME into adenosine, which inhibits the functions of various immune cells, such as DC and Tconv. Tregs also upregulate chemokines like CCL4, CCR5, CCR8, and CCR10, facilitating their accumulation in the tumor. In addition, Tregs overexpress several immune checkpoint molecules, including PD-1, LAG-3, TIM-3, TIGIT, and VISTA, which suppress anti-tumor effector cells. Treg also upregulate co-stimulatory molecules such as ICOS, GITR, and CD27, which enhance Treg activity and proliferation.

In summary, Tregs possess a variety of immunosuppressive mechanisms that can diminish the efficacy of anti-tumor therapies (**Figure 1**). Targeting these mechanisms opens up possibilities for developing strategies that could significantly improve anti-tumor treatments.

## 4 Targeting Ti-Tregs

### 4.1 Specific depletion or inhibition of Tregs

In peripheral blood, Treg cells account for 2-5% of all CD4+ T cells. While within tumor tissues, the Tregs proportion can reach up to 50%, and most of them are eTregs, which are crucial in establishing the immunosuppressive microenvironment of tumors (41). Specifically, eliminating or inhibition intratumoral eTreg cells can relieve the immunosuppression of the tumor microenvironment.

Tregs highly express molecules such as CTLA-4, CD25, PD-1, ICOS, LAG-3, TIGIT, GITR, 4-1BB, and OX40, which are ideal targets in immunotherapy (53). For example, the monoclonal antibody targeting the immune checkpoint CTLA-4, which has been used in the clinic, was primarily designed to block the immune checkpoint inhibitory signaling of effector T cells, thereby amplifying the Teff antitumor responses (128). Additionally, when antibody’s Fab region binds to the epitope on the surface of target cells, the Fc region recruits effector cells (such as NK cells and macrophages) and interacts with their Fc receptors (FcγR). This interaction mediates the direct killing of target cells by the effector cells, a process known as antibody-dependent cellular cytotoxicity (ADCC). Subsequent studies revealed that several CTLA-4 monoclonal antibodies can also eradicate Ti-Tregs via the ADCC effect, resulting in enhanced therapeutic outcomes (129–131). However, the efficacy of targeting CTLA-4 to deplete Tregs is still controversial. Some clinical studies have indicated that the number of Tregs within tumors does not decrease after CTLA-4 antibodies administration (132). It is also reported that CTLA-4 blocking antibody promoted the CD28-dependent expansion of Ti-Tregs (133). Additionally, as other immune cells within the microenvironment, such as activated Teffs and DCs (134), also express CTLA-4, nonselectively targeting CTLA-4 might inadvertently diminish antitumor immune cells. Thus, more detailed and comprehensive studies are still required to achieve favorable results with strategies targeting CTLA-4 to deplete Tregs.

Numerous studies have explored anti-tumor strategies targeting CD25 or combining IL-2 with cytotoxic molecules or CD25-ADC (antibody-drug conjugate) to deplete Treg cells. However, these approaches show variable effectiveness across different contexts (135–139). For instance, clinical trials using a fusion protein of IL-2 with diphtheria toxin (Denileukin Diftitox) for treating metastatic melanoma indicate that it cannot effectively eliminate intratumoral Treg cells, resulting in inadequate antitumor responses (136). Furthermore, the motifs in Denileukin Diftitox’s molecular structure may facilitate its binding to endothelial cells, resulting in endothelial damage and subsequent vascular leak syndrome, characterized by the leakage of plasma or proteins into surrounding tissues, leading to tissue edema, hypotension, and in severe cases, organ failure. This dose-limiting toxicity also restricts the anti-tumor application of this therapy (140). Since regular effector T cells also express the CD25 molecule, targeting CD25 could lead to the clearance of these conventional T cells. Some strategies involve optimizing the Fc segment of anti-CD25 monoclonal antibodies to favor binding with intratumoral FcγRIIb, specifically boosting the ADCC effect against intratumoral Tregs and promoting their elimination (141). Besides, employing a non-IL-2 blocking anti-CD25 antibody, Tregs can be eliminated while retaining the responsiveness of effector T cells to IL-2 signaling, thus amplifying the proliferation and function of anti-tumor T cells (142).

Similarly, other targets highly expressed on Treg cells, such as immune checkpoints LAG3, TIM3, VISTA and TIGIT, members of the tumor necrosis factor receptor superfamily (OX40, 4-1BB), immunoglobulins (ICOS, CD28) and G-protein coupled receptors, can all serve as potential targets for Treg cell elimination or inhibition. The combination of multi-target therapies might further enhance the efficacy of Treg-targeting treatment (143–145).

Anti-PD-1 therapy enhances the effector activity of Tconv cells and has shown promising outcomes in cancer treatment. However, it may also result in Treg activation and contribute to HPD (60, 63, 146). Studies have shown that in mouse models with a high PD-1+ Treg to PD-1+ CD8+ T cell ratio, PD-1 blockade could lead to tumor enlargement (147). Therefore, a combined approach targeting both PD-1 and other Treg markers to augment Teff activity while inhibiting Treg functions might be an effective strategy to enhance anti-tumor efficacy.

CTLA-4 and PD-1 are key immune checkpoints with distinct mechanisms in regulating T cell activity. CTLA-4 primarily functions during the immune priming phase, where its blockade enhances the activation and proliferation of Teffs as well as reducing Treg-mediated immune suppression. Conversely, PD-1 primarily functions during the effector phase, reversing exhausted T cell to an effector state (148). In melanoma patients with immune checkpoint blockade therapy, the peripheral blood CD8+ T cell TCR repertoire analysis provides insightful observations. Specifically, in the majority of patients (10 out of 12) who clinically benefited from PD-1 blockade therapy, there were no significant alterations in the frequency or diversity of circulating melanoma-reactive CD8+ T cell responses. However, the blockade of CTLA-4 resulted in a noticeable increase in the number of detectable melanoma-specific CD8+ T cell (149, 150). Theoretically, combining CTLA-4 and PD-1 blockade therapies is hypothesized to synergistically enhance anti-tumor effects. This has been confirmed in clinical settings, where combined therapy using ipilimumab (CTLA-4 antibody) and nivolumab (PD-1 antibody) has demonstrated greater efficacy than monotherapy in treating advanced melanoma (151). Numerous clinical trials are currently exploring this combination approach. However, it’s crucial to recognize that although the combination of CTLA-4 and PD-1 therapies can enhance effector T cell function and diminish Treg activity, it also disrupts immune homeostasis and leads to pronounced immune-related adverse events (irAEs), highlighting the necessity for diligent management and monitoring of patients receiving these combined treatments (152).

PD-1 blockade therapy has been shown to increase the expression of other immune checkpoints, such as TIM-3, Hammerman and colleagues discovered that prolonged exposure to PD-1 blockade antibodies escalates TIM-3 expression in non-small cell lung cancer patients (153, 154). Similarly, Xu et al. also found a significant increase in TIM3 expression within melanoma tumors of patients resistant to PD-1 treatment (155, 156). Preclinical models have confirmed that combining PD-1 and TIM-3 targeting in anti-tumor therapy is more effective than using either approach individually (154, 156).

Studies have constructed fusion proteins by combining PD-1 antibody and the GITR ligand (157). PD-1-GITR-L bispecific molecules were able to enhance the activation, proliferation, and memory formation of Tconvs *in vivo* and also boost the cytotoxicity of NK cells while reducing Tregs and exhausted T cells within the TME.

Studies have shown that increased TGF-β expression in tumors leads to an increase in PD-L1 positive cells within the TME, which, in turn, diminishes the effectiveness of PD-1/PD-L1 therapies (158). Further research found that the resistance to anti-PD-1 therapy in tumors correlates with increased TGF-β secretion by tumor fibroblasts and a decrease in CD8+ T cell infiltration (159). It has been established that inhibiting TGF-β signaling can significantly improve the efficacy of PD-1 antibody treatments (160). In addition, bispecific antibodies (TM101 and BiTP) targeting both TGF-β and PD-L1 have been shown to be more effective than anti-PD-L1 or anti-TGF-β monotherapy in various murine tumor models. This dual-targeting strategy can reduce collagen accumulation in tumors and promote T cell tumor infiltration, with great potential for application (161, 162).

In a preclinical model of head and neck squamous carcinoma, a VISTA blocking antibody monotherapy enabled the reversal of CD8+ T cell exhaustion into an effector state. However, it did not inhibit the recruitment of Tregs into the tumor and thus cannot effectively inhibit tumor growth. As CTLA-4 blockade significantly reduces Treg infiltration, the he combined administration of VISTA and CTLA-4 antibodies increases the CD8+T/Treg ratio and the CD4+Tconv/Treg ratio, significantly inhibiting tumor growth. Additionally, in prostate cancer patients treated with ipilimumab, a high expression of VISTA has been observed, indicating that the combined blockade of CTLA-4 and VISTA presents a promising strategy for enhancing anti-tumor efficacy (163).

### 4.2 Reduced recruitment of Tregs

Treg cells highly express certain chemokine receptors (CCR4, CCR5, CCR8, CCR10), which facilitates their preferential migration to the tumor microenvironment (164, 165). By inhibiting the chemokine signaling pathway, the accumulation of Tregs within tumors can be mitigated. For example, targeting CCR10 or CCR5 with receptor-blocking monoclonal antibodies significantly delayed tumor growth in mouse models (88, 166, 167). Moreover, certain monoclonal antibodies can also specifically eradicate Tregs through ADCC. Clinical trials with monoclonal antibodies against CCR4 have further validated their potential in depleting Treg cells (168, 169). Interestingly, tumors with naturally low expression of chemokines like CCL17/CCL22 demonstrate elevated chemokine expression following immune checkpoint blockade treatment (91). In such scenario, dual-targeting therapy involving chemokine receptors and immune checkpoints might aid in enhancing the efficacy of anti-tumor immunotherapy. In a clinical trial that combined mogamulizumab with nivolumab, 96 participants received dual-targeted therapy against CCR4 and PD-1, showing good safety profile and effectiveness in reducing eTregs and enhancing CD8+ T cell infiltration in tumors. Additionally, a study using an oral CCR4 antagonist combined with ICB (anti-CTLA-4 or anti-PD-1) therapy in a mouse tumor model demonstrated that the combination therapy is more effective against tumors than monotherapy (170, 171).

### 4.3 Attenuates the immunosuppressive function of Tregs

Treg cells, by highly expressing CD39 and CD73, adapt to the microenvironment and eventually convert intratumoral ATP to adenosine (104). Type 2 (P2) purinergic receptors, which recognize and bind to extracellular ATP, are categorized into two subtypes: P2X and P2Y. ATP, when acting on P2XR of Tregs, can induce Treg apoptosis and suppress Treg function (172, 173). CD39 reduces ATP levels in the TME, indirectly protecting Treg cells from ATP-triggered apoptosis. Meanwhile, CD73 elevates the adenosine concentration in the microenvironment. Adenosine binds to A2aR and A2bR on effector T cell membranes, suppressing their activity (106). Therefore, targeting CD39 and CD73 or using P2XR activators or antibodies blocking A2aR and A2bR can all alleviate the immunosuppressive effects of Tregs (107, 110).

Moreover, IDO acts as a tryptophan-consuming enzyme, leading to diminished levels of tryptophan within the TME. This amino acid is vital for the activation of T cells and their antitumor potentials (111). Notably, overexpression IDO in TME after immune checkpoint inhibitor administration, resulting in Tonvs dysfunction and heightened activation and proliferation of Tregs and MDSCs, establishing an immunosuppressive milieu (111, 174). Clinical trials have already been initiated for several IDO-targeting inhibitors (175).

Targeting molecules associated with Treg’s metabolic functions to reduce their activity offers a promising therapeutic strategy. For instance, Treg cells utilize the fatty acid transporter (CD36) and the lactic acid transporter MCT1 to transport free fatty acids and lactic acid from the glucose-deprived TME into cells, facilitating energy metabolism essential for their functionality (120–122). Studies targeting CD36 and MCT1 have demonstrated that focusing on these molecules can amplify the therapeutic effects of ICB treatments (120, 122).

In addition to targeting markers or key molecules highly expressed in Tregs, some studies have identified specific signaling pathways vital for Treg survival and functionality. Tregs with a specific deficiency or inhibition of the PI3Kδ molecule have restricted immunosuppressive capacities (176). Targeting PI3Kδ with small molecule inhibitors can diminish the Treg population within the TME, simultaneously boosting the activity of Teffs, reducing tumor progression, and suppressing tumor metastasis. Furthermore, Treg survival depends on sustained TCR activation signals, and there are differences in this signaling pathway between Tregs and Tconv cells (177, 178). Owing to the influence of the Foxp3 molecule, key components in this signaling pathway, such as LCK, ZAP70, and SLP76, are expressed at lower levels than in Tconvs, thus preventing apoptosis of T cells triggered by enduring TCR signal stimulation (179). Clinically, there are reports indicating that certain TKI inhibitors, like imatinib and dasatinib, due to their inhibitory effect on LCK, leading to a significant reduction in Treg cells (180, 181).

Targeting the VEGF-VEGFR pathway can also inhibit Treg cell functions and reduce their proportion in the TME. The monoclonal antibody against VEGFR2, ramucirumab, by blocking the VEGF-VEGFR2 signaling pathway, can decrease immunosuppressive cells such as Tregs, MDSCs, and M2 macrophages in the TME, while enhancing the presence of mature DCs (102, 182). Studies analyzing gastro-intestinal tumor samples pre- and postadministration of VEGFR2 monoclonal antibodies observed a notable reduction in eTreg cells within the TME (102). Additionally, after inhibiting the angiogenesis signaling pathway, there is a decline in the expression of effector T cell exhaustion markers (183). Clinically, there is a promising trend to co-administer antiangiogenic treatments with immune checkpoint inhibitors for enhanced antitumor therapy (184). Notably, the combination strategy achieves excellent results in treating hepatocellular carcinoma, renal cell carcinoma, non-small-cell lung cancer and colorectal cancer (185–188).

### 4.4 Treatment strategies modified based on kinetics and spatial distinction

While there are currently many treatment strategies for Tregs, few have yielded definitive results. One reason is that the characteristic molecules and metabolic pathways of Treg cells are not unique to themselves. Other antitumor effector cells within the immune system may also employ the same mechanisms for their function. Thus, a systemic drug delivery approach might result in the elimination or suppression of non-tumor Tregs outside the tumor as well as other effector cells. It may not only suppress pTreg functions, disrupting immunological homeostasis and triggering autoimmune inflammation, but also attenuate anti-tumor efficiency (189). Current research indicates that although certain cellular molecules are expressed in both Tregs and effector immune cells, there is a difference in their expression levels and kinetics. For instance, molecules like CD25 and CTLA-4 are consistently highly expressed in Tregs within tumors, while Tconv cells primarily express them at minimal levels in resting state but elevated levels upon activation (190).

Given the distinct expression patterns of CD25 between Tconvs and Tregs, the optimal timing for drug administration is chosen to specifically target Tregs while minimizing effects on Tconv. This concept was validated in clinical trials using CD25 monoclonal antibodies in conjunction with tumor antigen peptide vaccines, in which patients were administered a single dose of CD25 monoclonal antibody one week prior to vaccine treatment (135). That is, the drug clears CD25+ Tregs before antitumor-specific T cells are activated. This treatment effectively cleared Tregs and extended the progression-free survival of patients suffering from metastatic breast cancer. Clinical studies of anti-CTLA4 monoclonal antibodies along with tumor peptide vaccines also have proven that using CTLA-4 monotherapy before vaccination strengthens the antitumor reaction, enhancing therapeutic results (190).

Local drug delivery techniques can also be applied to improve treatment precision. for instance, utilizing interventional techniques to inject antibodies or drugs targeting Tregs locally or employing photodynamic drugs, coupling the photoactive dye IR700 with antibodies that target Tregs (191–193), and then irradiating the tumor site with near-infrared light, ensuring that the clearing antibodies locally exert their effects, leading to the suppression of both syngeneic and allogeneic tumors, even those untreated with near-infrared light.

## 5 Conclusion

This review encompasses a range of therapeutic strategies targeting Tregs for cancer treatment (**Table 1**). Given the varying roles and proportions of Tregs across different tumors, it is evident that no single Treg-targeting strategy can guarantee efficacy for every tumor type. Understanding the tumor immune microenvironment and selecting specialized therapeutic methods for Tregs is crucial. Moreover, the introduction of innovative drugs, together with comprehensive research on Treg subtypes and functions, promises significant advancements in Treg-targeted cancer therapies.

**Table 1:** Therapies targeting Tregs that are FDA approved or in clinical trials.

Therapeutic strategy	Target	Representative agents (drug type)	Clinical trial	Specific characteristics
Specific depletion of Treg	CD25 (IL-2Rα)	Daclizumab	FDA approved	Humanized anti-CD25 IgG1 blocking Ab
		Basiliximab	FDA approved	Recombinant chimeric (murine/human) anti-CD25 blocking mAb
		ADCT-301	NCT03621982, NCT02432235, NCT04052997, NCT04639024, NCT02588092	Anti-CD25 Ab (ADC conjugated)
		RO7296682	NCT04158583, NCT04642365, NCT05583617	Anti-CD25 Ab (IL-2 non-blocking Ab)
		RM-1995	NCT05220748	CD25-targeted near-infrared photoimmunotherapy
		Denileukin diftitox	FDA approved	IL-2-diphtheria toxin fusion protein
	CTLA-4	Ipilimumab	FDA approved	Humanized anti-CTLA-4 IgG1 blocking mAb
		ADU-1604	NCT03674502	
		Nurulimab	NCT03472027, NCT05751928, NCT05732805	
		Ticilimumab	FDA approved	Humanized anti-CTLA-4 IgG2 blocking mAb
		XMAB20717	NCT03517488	CTLA-4 and PD-1 bispecific mAb
		XMAB22841	NCT03849469, NCT05695898	CTLA-4 and LAG-3 bispecific mAb
		ATOR-1015	NCT03782467	CTLA-4 and OX40 bispecific mAb
	OX40	MEDI6383	NCT01862900, NCT02559024	Human OX40L-IgG4 Fc fusion protein
		PF-04518600	NCT03971409, NCT03390296	Agonistic anti-OX40 mAb
		BMS 986178	NCT03831295, NCT03410901, NCT02737475	
		BGB-A445	NCT04215978, NCT06029127, NCT05661955, NCT05635708	
		MEDI6469	NCT02559024, NCT02205333, NCT01862900, NCT02274155, NCT01303705	
		MOXR0916	NCT02410512, NCT02219724, NCT03029832	
		MEDI0562	NCT03336606, NCT02705482, NCT02318394, NCT03267589	
	GITR	MEDI1873	NCT02583165	Agonistic hexameric GITR ligand fusion protein
		TRX518	NCT02628574, NCT01239134, NCT03861403	Humanized, non-depleting, agylcosyl IgG1 mAb
		MK-1248	NCT02553499	Agonistic humanized IgG4 mAb
		BMS-986156	NCT04021043, NCT02598960	Agonistic humanized IgG1 mAb
		GWN323	NCT02740270	
	ICOS	JTX-2011	NCT02904226, NCT04319224, NCT03989362, NCT04549025	Agonistic humanized IgG1 mAb
		KY1044	NCT03829501	
		MEDI-570	NCT02520791	Humanized mAb with afucosylated Fc region
		GSK3359609	NCT02723955	Anti-ICOS Ab
		XMAB23104	NCT03752398, NCT05695898, NCT05879185	ICOS and PD-1 bispecific mAb
Reducing recruitment of Treg	CCR4	Mogamulizumab	FDA approved	Humanized anti-CCR4 mAb with afucosylated Fc region
		FLX475	NCT03674567, NCT04768686, NCT04894994	An orally accessible and specific CCR4 antagonist
	CCR8	GS-1811	NCT05007782	Humanized anti-CCR8 mAb with afucosylated Fc region
		BAY3375968	NCT05537740	
		BMS-986340	NCT04895709	
		S-531011	NCT05101070	Humanized anti-CCR8 mAb
Targeting intracellular signalling	PI3Kδ	Parsaclisib	NCT02646748	Small-molecule PI3Kδ inhibitor
		IOA-244	NCT04328844	
		AZD8186	NCT04001569, NCT04526470, NCT03218826, NCT01884285	
	LCK	Dasatinib	FDA approved	Multi-targeted TKIs
		Imatinib	FDA approved	
Targeting metabolic adaptation	CD36	VT1021	NCT03364400	Tsp-1 and lead to Treg aptosis
	MCT1	AZD3965	NCT01791595	MCT1 inhibitor
	IDO	Epacadostat	FDA approved	IDO inhibitor
		NLG802	NCT03164603, NCT05469490	
		GDC-0919	NCT02471846, NCT02048709, NCT05469490	
	CD73	LY3475070	NCT04148937	Small-molecule CD73 enzyme inhibitor
		CPI-006	NCT03454451	Anti-CD73 mAb
		MEDI9447	NCT03736473, NCT04262375	
		Sym024	NCT04672434	
	CD39	TTX-030	NCT04306900, NCT03884556	Anti-CD39 mAb
		SRF617	NCT04336098, NCT05177770	
Targeting the tumour microenvironment	VEGF	Bevacizumab	FDA approved	Anti-VEGF mAb
		Ramucirumab	FDA approved	Anti-VEGFR2 mAb
		Sorafenib	FDA approved	Multi-target small molecule inhibitors
	TGFβ	Galunisertib	NCT02423343	TGFR1 inhibitor
		M7824	FDA approved	Anti-PD-L1–TGFβ trap

*Tregs, regulatory T cells; IL-2, interleukin 2; CTLA4, cytotoxic T lymphocyte antigen 4; CCR,C-C chemokine receptor; GITR, glucocorticoid-induced TNFR-related protein; ICOS, integrated carbon observation system; PI3Kδ,phosphoinositide 3-kinase-δ; LCK, lymphocyte-specific protein tyrosine kinase; TKI, tyrosine kinase inhibitor; Tsp-1, stimulates thrombospondin-1; MCT1, monocarboxylate transporter 1; IDO, indoleamine 2,3-dioxygenase; VEGFR2, vascular endothelial growth factor receptor 2; TGFβ, transforming growth factor-β; TGFR1, TGF-β type I receptor; TNFR, tumor necrosis factor receptor.

## Author contributions

DQ: Writing – original draft. YZ: Writing – original draft. PS: Writing – original draft. YL: Writing – review & editing. XL: Writing – review & editing. YW: Project administration, Writing – review & editing.
